# Supplementary material for: Mycelium chemistry differs markedly between ectomycorrhizal and arbuscular mycorrhizal fungi
Source: Commun Biol. 2022 Apr 28;5:398. doi: 10.1038/s42003-022-03341-9 (PMC9050698; doi:10.1038/s42003-022-03341-9)
Supplement: Supplementary file 2 — Supplementary information [file 42003_2022_3341_MOESM2_ESM.pdf]

Supplementary Information

Mycelium chemistry differs markedly between ectomycorrhizal and arbuscular mycorrhizal fungi

Weilin Huang[1,2], Peter M. van Bodegom[1], Stéphane Declerck[3], Jussi Heinonsalo[4,5], Marco Cosme[3], Toni Viskari[4], Jari Liski[4], and Nadejda A. Soudzilovskaia[1,2]

[1]Environmental Biology, Institute of Environmental Sciences, Leiden University, Einsteinweg 2, 2333CC Leiden, the Netherlands.

[2]Centre for Environmental Sciences, Hasselt University, Martelarenlaan 42, 3500 Hasselt, Belgium.

[3]Mycology, Applied Microbiology, Earth and Life Institute, Université catholique de Louvain, 1348 Louvain-la-Neuve, Belgium.

[4]Climate System Research, Finnish Meteorological Institute, 00101 Helsinki, Finland.

[5]Department of Microbiology and Institute for Atmospheric and Earth System Research (INAR)/Forest Sciences, University of Helsinki, 00014 Helsinki, Finland.

Correspondence to: Weilin Huang (w.huang@cml.leidenuniv.nl)

Table S1. Samples of mycorrhizal fungi AM and EM with corresponding species names and CBS/MUL number (EM strains except Scleroderma verrucosum are all from CBS-KNAW collection, AM strains all from BBCM/MUCL). Single quotes' remarks for samples taken average values with multiple measurements. Star remarks* are the calculation results for samples being tested with a mixture of standard litter. Medium recipes of EMF are according to the manual of Westerdijk Fungal Biodiversity Institue[1].

**Sample**		**Name**	**CBS/MUL NO.**	**Medium**	**Drying Method**
EMF	EM-1	*Xerocomus rubellus*	CBS 230.66	CHA4.7	Freeze Dryer
	EM-2'	*Paxillus involutus*	CBS 100140	MOD	Freeze Dryer
	EM-3'	*Laccaria bicolor*	CBS 445.79	CHA	Oven 55℃ for 12 hours
	EM-4	*Inocybe rimosa*	CBS 210.55	X agar	Oven 55℃ for 12 hours
	EM-5'	*Hebeloma hiemale*	CBS 376.89	MEA	Oven 55℃ for 12 hours
	EM-6	*Lactarius deliciosus*	CBS 582.63	BAF	Freeze Dryer
	EM-7	*Phaeogyroporus sudanicus*	CBS 481.89	MMN/MOD	Freeze Dryer
	EM-8	*Peziza varia*	CBS 115948	OA	Freeze Dryer
	EM-9	*Cortinarius cristallinus*	CBS 348.74	MMN	Freeze Dryer
	EM-10	*Peziza quelepidotia*	CBS 135943	MYA/OA	Freeze Dryer
	EM-11*	*Scleroderma verrucosum*	MUCL34674	MMN	Oven 55℃ for 12 hours
AMF	AMF-1	*Rhizohpagus clarus*	MUCL46238	MSR	Freeze Dryer
	AMF-2	*Rhizohpagus irregularis*	MUCL41833	MSR	Freeze Dryer
	AMF-3*	*Glomus aggregatum*	MUCL49408	MSR	Freeze Dryer
	AMF-4	*Glomus hoi*	MUCL45686	MSR	Freeze Dryer
Supple-ments	Root_AMF*	Root tissue (*D.carota*) inoculated with AMF	-		Freeze Dryer
	Media-1'	Media for AMF	-	A mixture of AMF medium	Oven 55℃ for 12 hours
	Media-2	Media for EMF	-	A mixture of EMF medium	Oven 55℃ for 12 hours

Table S2. Chemical compositions of AM and EM fungi samples.

**Sample**		**Name**	**Fraction E**	**Fracion W**	**Fraction A**	**Fraction N**
EMF	EM-1	*Xerocomus rubellus*	14.49	42.16	38.79	4.56
	EM-2'	*Paxillus involutus*	10.00	31.16	52.94	5.90
	EM-3'	*Laccaria bicolor*	11.53	31.61	53.40	3.46
	EM-4	*Inocybe rimosa*	14.65	40.13	42.88	2.34
	EM-5'	*Hebeloma hiemale*	15.00	42.84	36.00	6.15
	EM-6	*Lactarius deliciosus*	15.02	40.62	41.49	2.87
	EM-7	*Phaeogyroporus sudanicus*	10.71	48.54	35.09	5.67
	EM-8	*Peziza varia*	14.30	37.29	46.86	1.55
	EM-9	*Cortinarius cristallinus*	16.26	40.23	40.74	2.77
	EM-10	*Peziza quelepidotia*	28.55	29.73	39.26	2.45
	EM-11*	*Scleroderma verrucosum*	18.10	16.90	48.89	16.11
AMF	AMF-1	*Rhizohpagus clarus*	8.15	18.47	72.65	0.73
	AMF-2	*Rhizohpagus irregularis*	7.89	18.48	72.61	1.02
	AMF-3*	*Glomus aggregatum*	11.59	15.95	61.46	11.00
	AMF-4	*Glomus hoi*	8.54	19.97	70.71	0.78
Supple-ments	Root_AMF*	*Root tissue (D.carota) inoculated with AMF*	10.00	17.46	67.57	4.97
	Media-1'	*Media for AMF*	7.98	13.61	77.90	0.51
	Media-2	*Media for EMF*	0.16	96.42	0.00	0.00

Table S3. PerMANOVA results of chemical composition between mycorrhizal groups. 'Df' represent degrees of freedom; 'Sum Sq' represent sum of squares; 'Mean Sqs' represent mean of squares; 'Pseudo-F' represent F value by permutation. The P value <0.05 indicates statistical significance, based on 999 permutations, using Bray-Curtis method.

	Df	Sum Sq	Mean Sqs	Pseudo-F	R^2	P
Strain	1	0.20063	0.200634	20.949	0.61708	0.001
Residuals	13	0.1245	0.009577		0.38292	
Total	14	0.32514			1	

Table S4. WAEN chemical compositions of plant foliage litter obtained from CIDET and LIDET database.

SPECIES	W	A	E	N	Plantgrowth
Abies amabilis	31.36	29.18	13.21	26.26	Evergreen Tree
Abies balsamea	27.27	31.07	13.62	28.05	Evergreen Tree
Abies concolor	39.42	35.66	17.79	7.13	Evergreen Tree
Abies lasiocarpa	35.92	26.73	14.70	22.65	Evergreen Tree
Abronia latifolia	31.96	30.65	19.43	17.96	Herb
Acer saccharum	41.58	34.60	9.00	14.82	Deciduous Tree
Alnus rugosa	32.03	38.48	8.50	20.99	Deciduous Tree
Ammophila breviligulata	21.72	57.34	6.45	14.50	Herb
Betula lutea(alleghaniensis)	18.73	46.28	8.00	26.99	Deciduous Tree
Betula papyrifera	37.15	31.35	6.74	24.77	Deciduous Tree
Bouteloua eriopoda	13.44	65.66	5.13	15.77	Herb
Bouteloua gracilis	14.16	70.12	7.62	8.11	Herb
Ceanothus greggii	49.60	27.11	10.79	12.50	Evergreen Shrub
Chamaecyparis nootkatensis	26.50	33.10	16.58	23.83	Evergreen Tree
Cornus nuttallii	52.47	37.57	9.19	0.77	Deciduous Tree
Drypetes glauca	40.67	40.28	8.05	11.00	Evergreen Tree
Epilobium angustifolium	61.77	27.31	8.39	2.53	Herb
Fagus grandifolia	15.14	49.13	7.56	28.16	Deciduous Tree
Festuca hallii	14.05	63.87	9.89	12.19	Herb
Gaultheria shallon	27.53	36.28	8.53	27.67	Evergreen Shrub
Gymnanthes lucida	40.50	42.90	9.87	6.80	Evergreen Tree
Kobresia myosuroides	22.99	62.27	5.38	9.35	Herb
Larix laricina	31.16	30.95	11.55	26.34	Deciduous Tree
Larix occidentalis	34.10	34.54	10.10	21.27	Evergreen Tree
Larrea tridentata	32.05	41.15	18.73	8.07	Evergreen Shrub
Liriodendron tulipifera	44.83	32.17	14.20	8.81	Deciduous Tree
Myrica cerifera	29.64	30.44	12.98	26.94	Evergreen Shrub
Picea abies	16.15	45.77	4.96	33.12	Evergreen Tree
Picea engelmannii	37.35	29.69	14.32	18.65	Evergreen Tree
Picea glauca	31.77	37.11	8.69	22.44	Evergreen Tree
Picea mariana	29.72	34.49	12.81	22.98	Evergreen Tree
Picea sitchensis	20.30	40.44	10.63	28.63	Evergreen Tree
Pinus banksiana	15.64	43.56	7.15	33.66	Evergreen Tree
Pinus contorta	18.83	40.89	10.58	29.69	Evergreen Tree
Pinus elliottii	19.68	41.46	17.37	21.50	Evergreen Tree
Pinus monticola	32.60	33.66	9.10	24.65	Evergreen Tree
Pinus ponderosa	27.38	32.49	11.59	28.54	Evergreen Tree

Pinus resinosa	18.46	42.74	14.81	23.99	Evergreen Tree
Pinus strobus	20.30	40.00	18.90	20.80	Evergreen Tree
Pinus sylvestris	15.50	49.10	10.62	24.78	Evergreen Tree
Populus tremuloides	33.65	38.99	10.05	17.31	Deciduous Tree
Pseudotsuga menziesii	18.59	40.41	12.38	28.62	Evergreen Tree
Quercus ellipsoidalis	25.90	41.20	7.40	25.60	Deciduous Tree
Quercus prinus	27.38	39.61	9.38	23.64	Deciduous Tree
Quercus rubra	24.48	40.93	5.84	28.76	Deciduous Tree
Rhododendron macrophyllum	36.63	37.27	9.00	17.11	Evergreen Shrub
Robinia pseudoacacia	34.12	40.78	7.23	17.88	Deciduous Tree
Schizachyrium gerardi	14.94	60.30	5.95	18.81	Herb
Schizachyrium scoparium	9.13	69.05	3.79	18.03	Herb
Spartina alterniflora	27.36	60.46	4.95	7.24	Herb
Taxus brevifolia	16.50	32.46	9.80	41.25	Evergreen Tree
Thuja occidentalis	17.66	39.88	11.92	30.55	Evergreen Tree
Thuja plicata	18.78	38.36	13.06	29.80	Evergreen Tree
Triticum aestivum	6.76	73.59	3.36	16.29	Herb
Tsuga heterophylla	14.78	47.84	5.18	32.21	Evergreen Tree
Tsuga mertensiana	26.77	29.93	9.47	33.82	Evergreen Tree
Vochysia ferraggenea	30.77	43.57	7.47	18.20	Evergreen Tree

Table S5. Effect sizes of chemical compositions differences among plant litters and mycorrhizal fungi.

	**Eta2**		**Omega2**		**Epsilon2**		**Chen's f**	
	Mycorrhiza fungi	Plant leaf	Mycorrhiza fungi	Plant leaf	Mycorrhiza fungi	Plant leaf	Mycorrhiza fungi	Plant leaf
**Water-soluble**	0.68	0.21	0.64	0.16	0.66	0.16	1.47	0.51
**Acid-hydrolysable**	0.76	0.41	0.73	0.38	0.74	0.38	1.77	0.84
**Ethanol-soluble**	0.46	0.28	0.4	0.23	0.41	0.24	0.92	0.62
**Non-hydrolysable**	0.26	0.20	0.2	0.15	0.21	0.15	0.6	0.49

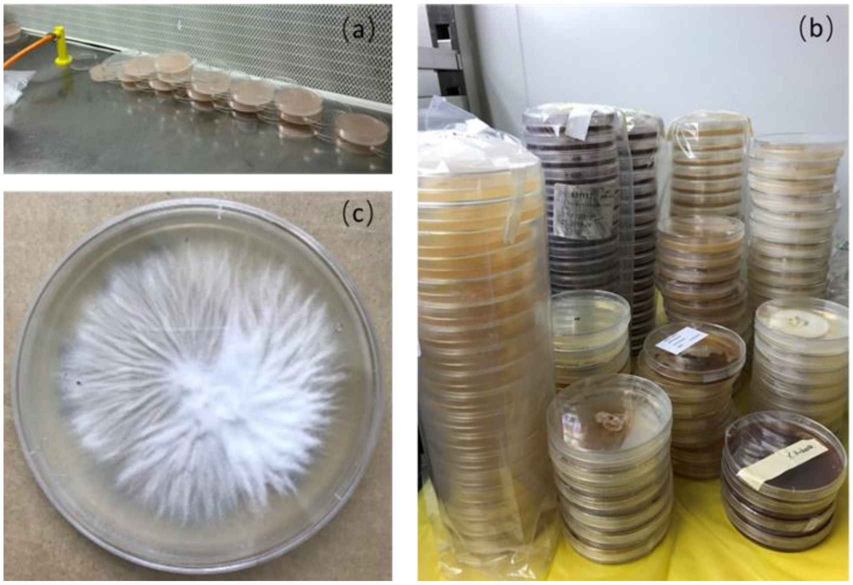

Fig.S1 In vitro cultivation of ectomycorrhizal fungi. (a) Medium preparation and inoculation under sterile conditions using laminar flow hood; (b) Inoculated EM fungi plates sealed and cultivated in the dark; (c) *Laccaria bicolor* cultivation on medium after 3weeks incubation in the dark (27℃).

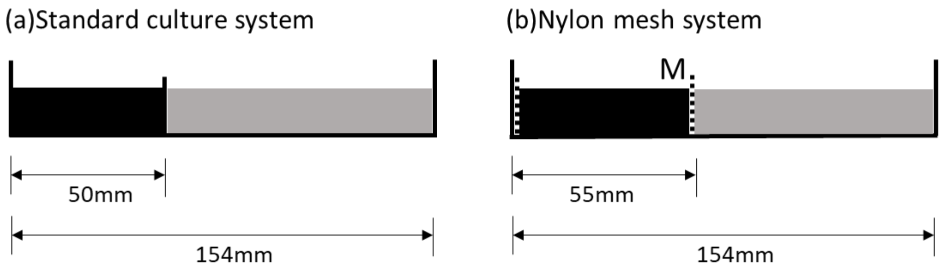

Fig.S2 Schematic representation of two physically different autotrophic whole plant systems, S1 and S2. The plant compartment is coloured black, the fungal compartment with the medium is coloured grey, the nylon mesh (M) is depicted as dashed line: (a) autotrophic whole plants system with a Petri (S1) root compartment; (b) autotrophic whole plants system with a mesh (S2) root compartment.

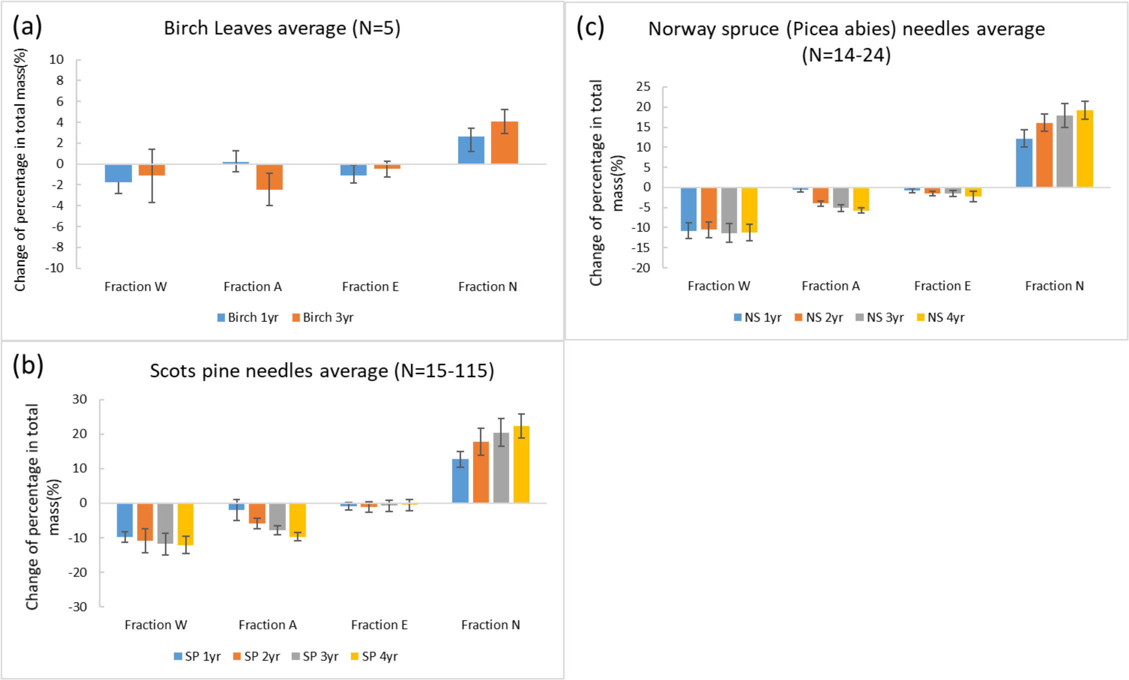

Fig.S3 Chemical composition variation in decomposing plant litter. (a) Chemical composition of WAEN in birch leaves (n=5) within 3 years, data from FMI litter decomposition experiment; (b) and (c), chemical composition of WAEN in needles of spruce (n=14-24) and pine (n=15-115) within 4 years decomposition, data from ED dataset.

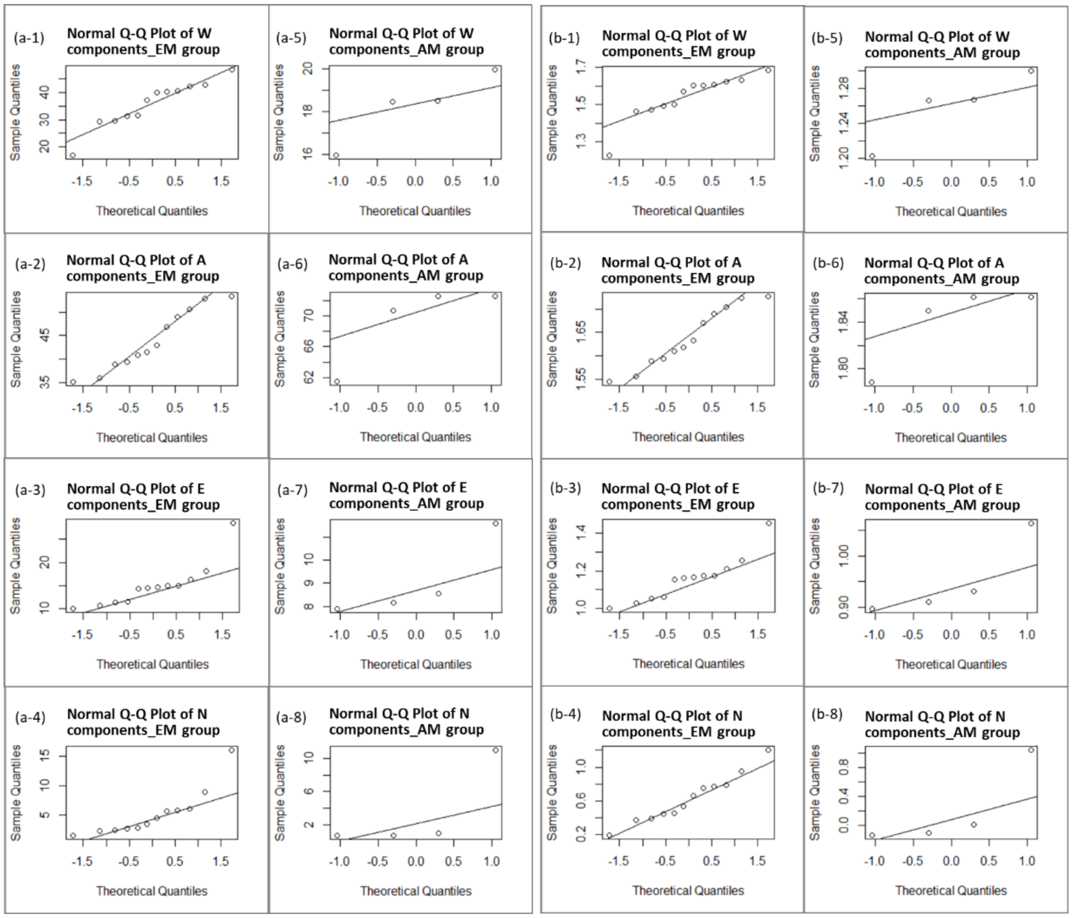

Fig.S4 Distribution of each chemical composition data of AM and EM sample groups. (a) Original data (%); (b) Data were log(x+1) transformed.

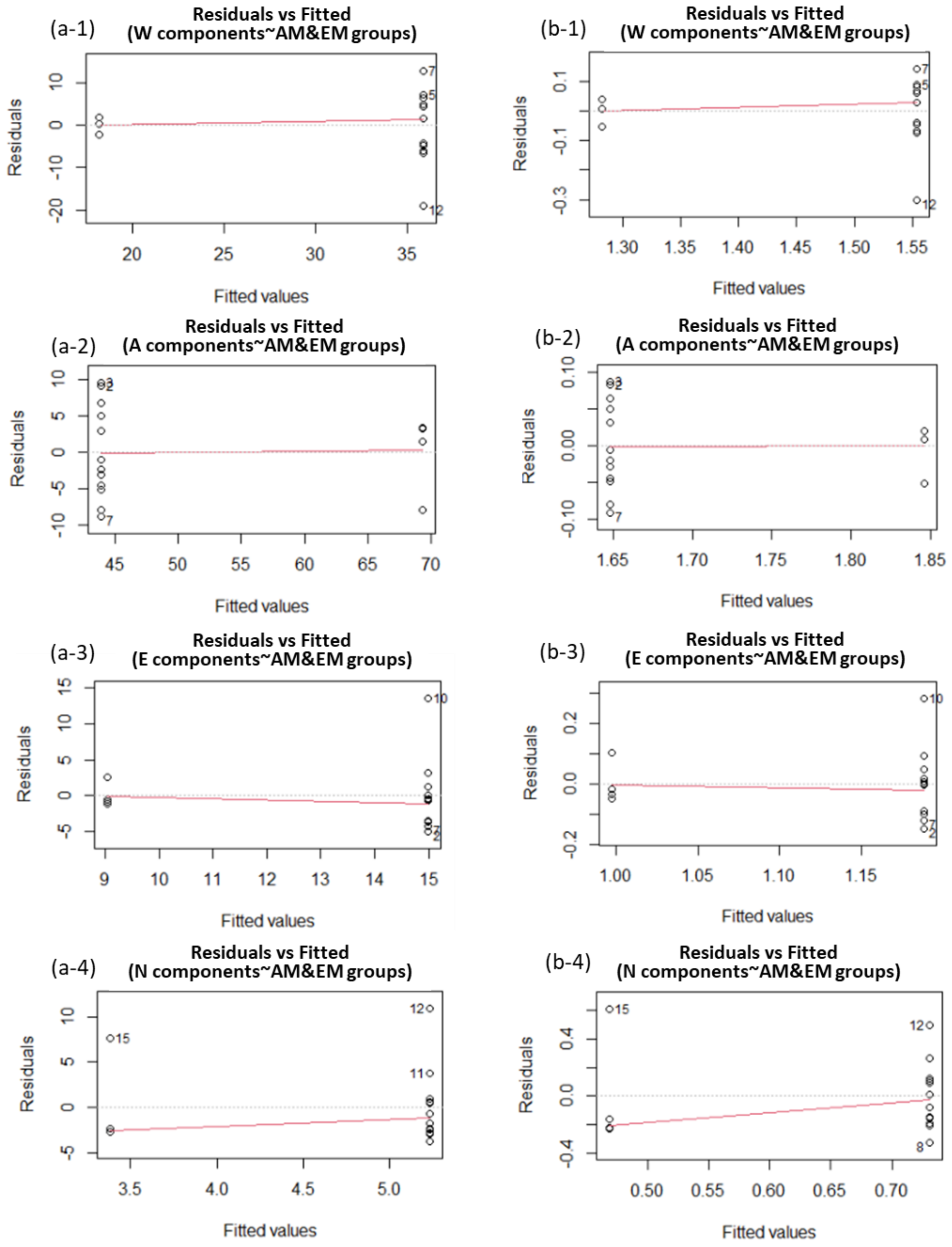

Fig.S5 Homogeneity of variance of chemical composition data of AM and EM sample groups. (a) Original data of AM and EM sample groups; (b) Log-transformed data of AM and EM sample groups.

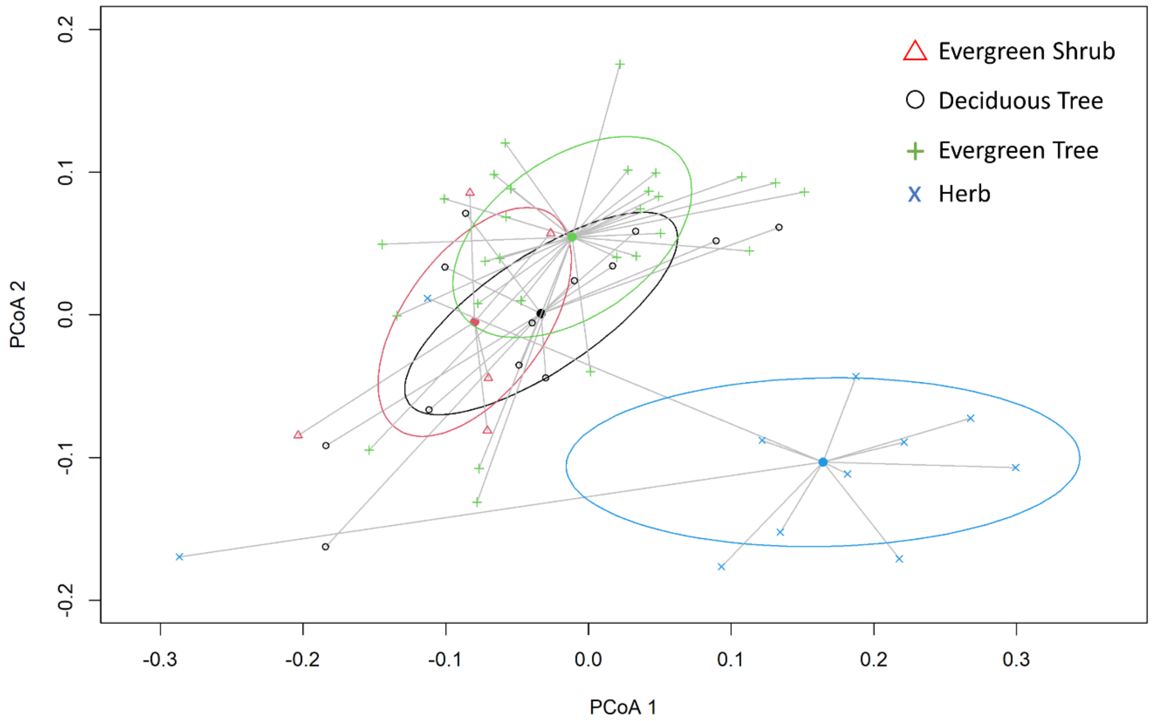

Fig.S6 Clustering and centroids of four plant functional types in a multidimensional space of WAEN components.

Notes S1. Plant litter data and statistics

We gathered plant leaf WAEN chemical composition data of 57 plant species from CIDET and LIDET datasets (Table S4). Data were averaged for species with multiple available measurements. Species were grouped into four functional types: evergreen tree, deciduous tree, evergreen shrub and herb, based on plant growth form information obtained from the TRY database[2]. Species with multiple growth form definitions were defined according to the highest occurrence frequency[3]. Following the ANOVA analysis to assess differences among plant functional types in WAEN parameters, we calculated effect size indices. This allowed us to compare the magnitude of the differences among WAEN of plants that belong to distinct PFTs to the differences among WAEN of AMF and EMF. As distinct effect size indices may yield different results, we opted to calculate four distinct indices: Eta2, Omega2, Epsilon2 and Chen's f (Table S5). Comparison across all indices revealed that the differences in magnitude of difference in WAEN among vascular plants and mycorrhizal fungi are highly consistent, with effect sizes of differences among mycorrhizal guilds being always 1.5 to 4 times higher than the effect size of differences among plant functional types.

Besides, beta-dispersion was calculated to test if the dispersions, variances of the litter from different plant growth forms are significantly different. An ANOVA was performed to evaluate difference in group dispersions within the dataset of plant litter. The results ($p=0.714$, $F=0.714$, $df=3$) shows that the group dispersions, or variances, are not significantly different from each other and the group dispersions are likely homogeneous between groups. We plot the dispersion for each group as shown in Fig.S6. As expected, since we have identified no significant differences, the

deciduous and evergreen groups have overlaps and are not distinct from each other. However, with further perMANOVA test, we found that even groups in plant litter have similar dispersions, their centroids are still significantly different (R^2=0.311, p=0.001, df=3).

Supplementary References

1. Crous, P. W., Verkley, G. J. M., Groenewald, J. Z. & Houbraken, J. *Westerdijk Laboratory Manual Series 1: Fungal Biodiversity.* (2019).
2. Kattge, J. *et al.* TRY - a global database of plant traits. *Glob. Chang. Biol.* **17**, 2905–2935 (2011).
3. Engemann, K. *et al.* A plant growth form dataset for the New World. *Ecology* **97**, 3243 (2016).
