# Supplementary material for: Mycelium chemistry differs markedly between ectomycorrhizal and arbuscular mycorrhizal fungi
Source: Commun Biol. 2022 Apr 28;5:398. doi: 10.1038/s42003-022-03341-9 (PMC9050698; doi:10.1038/s42003-022-03341-9)
Supplement: Supplementary file 3 — Reporting Summary [file 42003_2022_3341_MOESM3_ESM.pdf]

nature portfolio

Corresponding author(s): Weilin Huang

Last updated by author(s): Feb 28, 2022

Reporting Summary

Nature Portfolio wishes to improve the reproducibility of the work that we publish. This form provides structure for consistency and transparency in reporting. For further information on Nature Portfolio policies, see our Editorial Policies and the Editorial Policy Checklist.

Statistics

For all statistical analyses, confirm that the following items are present in the figure legend, table legend, main text, or Methods section.

n/a	Confirmed	
☐	☒	The exact sample size (n) for each experimental group/condition, given as a discrete number and unit of measurement
☐	☒	A statement on whether measurements were taken from distinct samples or whether the same sample was measured repeatedly
☐	☒	The statistical test(s) used AND whether they are one- or two-sided<br>*Only common tests should be described solely by name; describe more complex techniques in the Methods section.*
☐	☒	A description of all covariates tested
☐	☒	A description of any assumptions or corrections, such as tests of normality and adjustment for multiple comparisons
☒	☐	A full description of the statistical parameters including central tendency (e.g. means) or other basic estimates (e.g. regression coefficient) AND variation (e.g. standard deviation) or associated estimates of uncertainty (e.g. confidence intervals)
☐	☒	For null hypothesis testing, the test statistic (e.g. F, t, r) with confidence intervals, effect sizes, degrees of freedom and P value noted<br>*Give P values as exact values whenever suitable.*
☒	☐	For Bayesian analysis, information on the choice of priors and Markov chain Monte Carlo settings
☒	☐	For hierarchical and complex designs, identification of the appropriate level for tests and full reporting of outcomes
☐	☒	Estimates of effect sizes (e.g. Cohen's d, Pearson's r), indicating how they were calculated

Our web collection on statistics for biologists contains articles on many of the points above.

Software and code

Policy information about availability of computer code

Data collection	No software was used
Data analysis	Data were analyzed in R code, the packages used have been explained in Methods section.

For manuscripts utilizing custom algorithms or software that are central to the research but not yet described in published literature, software must be made available to editors and reviewers. We strongly encourage code deposition in a community repository (e.g. GitHub). See the Nature Portfolio guidelines for submitting code & software for further information.

Data

Policy information about availability of data

All manuscripts must include a data availability statement. This statement should provide the following information, where applicable:

- Accession codes, unique identifiers, or web links for publicly available datasets
- A description of any restrictions on data availability
- For clinical datasets or third party data, please ensure that the statement adheres to our policy

All data generated or analyzed during this study are included in this article (and its supplementary information files).

Field-specific reporting

Please select the one below that is the best fit for your research. If you are not sure, read the appropriate sections before making your selection.

☐ Life sciences ☐ Behavioural & social sciences ☒ Ecological, evolutionary & environmental sciences

For a reference copy of the document with all sections, see nature.com/documents/nr-reporting-summary-flat.pdf

Ecological, evolutionary & environmental sciences study design

All studies must disclose on these points even when the disclosure is negative.

Study description	This study looks into the mycelium chemical quality of two dominant mycorrhizal types. We selected 4 species of AM fungi and 11 species of EM fungi representing the diversity of these two mycorrhiza types. Mycelium biomass was gathered from multiple in vitro cultivation plates to characterize its chemical composition. Samples of different species were treated as replicates of the corresponding mycorrhizal type.
Research sample	EM fungal species are Xerocomus rubellus, Paxillus involutus, Laccaria bicolor, Inocybe rimosa, Hebeloma hiemale, Lactarius deliciosus, Phaeogyroporus sudanicus, Peziza varia, Cortinarius cristallinus, Peziza quelepidotia, and Scleroderma verrucosum; AM fungal species are Rhizophagus clarus, Rhizophagus irregularis, Glomus aggregatum and Glomus hoi.
Sampling strategy	We selected available AM fungal species strains from the Glomeraceae family, as this family is globally the most dominant family of AM fungi, while they can be grown in vitro producing reasonably large amounts of fungal biomass. We selected EM fungal species to cover relatively abundant strains of various families. In addition, we opted to use a higher number of EM fungi species compared to AM fungi, because (1) EM fungi consist of ca. 20,000-25,000 species, which likely entail a high diversity of chemical traits, while AM fungi have been known to exhibit lower diversity with ca. 300 species identified within this fungal phylum, (2) mass-production of AM fungi to reach the amounts of biomass necessary for the chemical composition assessment is complicated, necessitating hundreds of Petri plates.
Data collection	Mycelia from cultivation each species were dried and stored in frozen before chemical testing. Samples were examined for the chemical composition by the technician in the laboratory of Natural Resources Institute (Finland). Then J.H. processed the initial data and shared the results with co-authors.
Timing and spatial scale	EM fungi samples were collected with a cultivation period of 4-5 weeks depending on the growth of individual species. AM fungi have longer cultivation periods, normally taking 4-5 months before being ready for the first harvest depending on species, system types and plates conditions.
Data exclusions	No data was excluded.
Reproducibility	We did multiple analyses for the same species of EM fungi Hebeloma hiemale, and conducted accuracy assessment based on that. All attempts are successful.
Randomization	The mycelium data were grouped by the mycorrhiza type. Covariates were not relevant to this study as all mycorrhiza were grown at controlled and constant conditions. However, during the chemical composition analysis, samples were randomized to avoid non-random technical deviations to affect our results.
Blinding	The samples were tested in a third party lab, with only sample numbers to eliminate experimental biases that arise from participants' expectations.

Did the study involve field work? ☐ Yes ☒ No

Reporting for specific materials, systems and methods

We require information from authors about some types of materials, experimental systems and methods used in many studies. Here, indicate whether each material, system or method listed is relevant to your study. If you are not sure if a list item applies to your research, read the appropriate section before selecting a response.

Materials & experimental systems

n/a	Involved in the study
☒	☐ Antibodies
☒	☐ Eukaryotic cell lines
☒	☐ Palaeontology and archaeology
☒	☐ Animals and other organisms
☒	☐ Human research participants
☒	☐ Clinical data
☒	☐ Dual use research of concern

Methods

n/a	Involved in the study
☒	☐ ChIP-seq
☒	☐ Flow cytometry
☒	☐ MRI-based neuroimaging
